# A family perspective for the mechanism of parent-child conflict on maternal anxiety in Chinese children with autism

**DOI:** 10.1186/s40359-024-01786-7

**Published:** 2024-05-22

**Authors:** Xue Du, Le Sun, Qi Dong

**Affiliations:** 1 College of Educational Science, Chongqing Normal University, Chongqing, 401331 China; 2 Key Laboratory of Applied Psychology, Chongqing Normal University, Chongqing, 401331 China

**Keywords:** Mothers of children with autism, Anxiety, Child problem behavior, Parent-child conflict, Parenting stress

## Abstract

**Background:**

Mothers of children with autism reported higher levels of anxiety than mothers of typical children. This study revealed the relationship between parent-child conflict, children’s problem behavior, parenting stress, and maternal anxiety from the perspective of the relationship within the family.

**Methods:**

The State-Trait Anxiety Inventory (STAI) and Caregiver Strain Questionnaire (CGSQ) were used to measure maternal anxiety and parenting stress respectively from 102 mothers of children with autism. We also collected information on parent-child relationships and children’s problem behaviors by using the Child-Parent Relationship Scale (CPRS) and Conners Parent Symptom Questionnaire (PSQ).

**Results:**

Parent-child conflict positively predicted state and trait anxiety in mothers of children with autism. The severity of children’s psychosomatic disorders fully mediated the positive association between parent-child conflict and state-trait anxiety in mothers of children with autism. Parenting stress significantly moderated the impact of parent-child conflict on maternal state anxiety and trait anxiety.

**Conclusion:**

In the case of children with autism spectrum disorders, parent-child conflict can directly affect maternal anxiety levels, especially when mothers have low levels of parenting stress. Parent-child conflict can also affect children’s problem behaviors and thus indirectly affect maternal anxiety. Therefore, this study is of great significance for the alleviation of anxiety of mothers of autistic children and the family intervention for the early rehabilitation of autistic children.

## Introduction

Autism spectrum disorder (ASD) is a neurological disorder characterized by difficulties in social interactions, patterns of communication, and repetitive behaviors and/or interests, which can lead to difficulties with extensive social interaction, communication, and participation in daily activities [1]. Recently, the incidence of autism in the United States was reported as high as 1 in 36 children [2]. The development and adaptation of children with ASD are difficult to understand and unpredictable, which can easily lead to uncertainty in mothers [3]. Therefore, parenting a child with ASD presents unique challenges to parents that can negatively impact caregivers’ mental health [4]. As the child’s primary caregiver, mothers of children with ASD were at increased risk for anxiety compared with fathers [5], healthy children, or children with other developmental disabilities [6–9].

Anxiety is an unpleasant state of fear and anxiety, accompanied by physical activation. It often involves efforts and expectations to avoid danger and threat, but inefficient in dealing with that danger or threat [10]. Anxiety is divided into two types: State-anxiety and Trait-anxiety [11]. State anxiety refers to a person’s temporary, passive state. This state exists immediately and has a certain intensity level. Excessive state anxiety levels will damage an individual’s mental health. Trait anxiety refers to stable individual differences in a person’s relatively enduring personality characteristics [12].

Ecosystem theory believes that the family microsystem is an important component of the ecosystem that affects the development of family members [13]. As two important family micro factors, parent-child relationship, and parenting pressure have a profound impact on individual physical and mental development [13–15]. For example, a good parent-child interaction can reduce the mother’s depression and anxiety [16], while a poor parent-child relationship can lead to the individual suffering from mental illness [17, 18], such as depression and anxiety [19]. Parent-child conflict, as a manifestation of a negative parent-child relationship [20, 21], can increase the mother’s parenting difficulty leading to more anxiety in the typical development populations. However, the relationship between the parent-child relationship and anxiety in Chinese mothers of children with ASD is unclear.

Similarly, according to the transactional models [22], parental characteristics can not only affect children’s development outcomes, but children’s development outcomes can also affect parents’ emotional health levels and behaviors [23, 24], there is a continuous interaction between children and their mothers [25–27]. Previous results have confirmed that compared with children with low behavioral problems, parents of children with high behavioral problems have higher anxiety levels [28] and parents’ trait anxiety can positively predict preschool children’s emotional problems [29]. That is to say, children’s problem behaviors caused by diseases are an important source of stress and depressive symptoms for parents of children with ASD [30–32], which harms the mother’s health [33].

To sum up, according to the family systems theory, children’s problem behavior, parenting stress, and parent-child conflict may work together on anxiety, but few studies have focused on mothers of children with ASD. Therefore, this study will start from the main dimension of maternal anxiety of ASD children to explore the relationship between the parent-child relationship and ASD maternal anxiety, as well as the possible mechanisms of children’s problem behaviors and parenting stress. This can help us better understand the role of family factors in the onset of autism, improve the quality of life of families of children with autism, and promote early intervention of children with autism.

## Method

### Participants

In this study, a questionnaire survey was used to collect hypothesis test data. The formal investigation was conducted from February to June 2023 at designated disabled Persons federations and special education institutions in Chongqing. We used G-Power3.1 to measure the sample size we needed, with 108 participants needed to achieve 80% statistical test power at a moderate effect size (F = 0.25) and a significance level of 0. 05. The survey was conducted online and offline at the same time, a total of 118 data were received, and 102 were finally received, with a recovery rate of 86%. Factors such as incomplete paper, online recycling, and short or long regular response time were excluded. Inclusion criteria for children with autism: (1) Meet the diagnostic criteria of the American Diagnostic and Statistical Manual of Psychiatry, Fifth Edition (DSM-5); (2) The family members of the children signed a written informed consent form. Inclusion criteria for mothers of children with ASD: (1) Mothers of children with ASD who have been diagnosed; (2) Clear consciousness, no intellectual disability, and able to complete the scale assessment; (3) Voluntarily participate in the survey. The study obtained the informed consent of all participating mothers, and mothers were informed of the ethical principles of voluntary participation. This study was in line with the Declaration of Helsinki and approved by the Ethics Committee of the College of Educational Sciences, Chongqing Normal University.

### Research tools

#### Demographic information

A self-made questionnaire was used to collect the demographic information of the research subjects, including the mother’s age, marital status, education, occupations, family income, number of children, children’s age and gender, cost of treatment (see Table 1).

#### State-trait anxiety inventory (STAI)

The State-Trait Anxiety Inventory developed by Spielberger was used to measure the anxiety level of the participants [34]. The Chinese revised version of the measurement tool used in this study contains two subscales (40 items in total). Questions 1 to 20 are the State Anxiety Inventory. Questions 21 to 40 were the Trait Anxiety Inventory [35]. Both the state anxiety and trait anxiety subscales were scored on a scale of 1 to 4. The higher the cumulative total score, the higher the individual’s state anxiety level or the more obvious the trait anxiety [36]. In this study, the Cronbach’s α coefficient of this scale was 0.905.

#### Child-parent relationship scale (CPRS)

The Child-Parent Relationship Scale was compiled by Pianta to investigate the parent-child relationship in families with young children [37, 38]. Two dimensions including parent-child intimacy and parent-child conflict with a total of 22 questions in this scale. Participants were asked to answer on a 5-point Likert scale ranging from “completely inconsistent to completely consistent.” Two dimensions are scored separately, the higher the score, the higher the conflict or intimacy between parents and children. In this study, a parent-child conflict subscale was selected to measure the negative conflict between mother and child. The final score was calculated by averaging all the questions, and the higher the score, the higher the conflict between parents and children. The Cronbach’s α coefficient for this dimension was 0.879.

#### Caregiver strain questionnaire (CGSQ)

The Caregiver Strain Questionnaire was compiled by Brannan and introduced to China by the Institute of Mental Health of Peking University in 2001, it had good test-retest reliability and structural validity [39, 40]. The Caregiver Stress Questionnaire assessed the caregiver’s parenting stress, with a total of 21 items, including 3 dimensions, namely objective pressure, subjective internal pressure, and subjective external pressure. It adopted a five-level score of 1 to 5 points. The higher the score, the higher the caregiver’s stress [41]. Another study found that it had good reliability and validity in assessing the stress issues of caregivers of autistic children [42]. In this study, the Cronbach’s α coefficient of this scale was 0.945.

#### Conners parent Symptom Questionnaire (PSQ)

The Conners Parent Symptom Questionnaire was compiled by Conners in 1969, this scale was widely used abroad and had good reliability and validity [43]. It was also suitable for assessing behavioral problems of Chinese children aged 3 to 17 years old [44]. The scale consists of 48 questions, including five dimensions: conduct problems, learning problems, psychosomatic disorders, impulsive-hyperactive and anxiety. There were 4 levels of scoring, with options ranging from 0 to 3, representing “none” to “a lot” respectively. The questionnaire was filled out by the child’s father or mother, with higher scores indicating more severe behavioral problems. In this study, the Cronbach’s α coefficient of this scale was 0.962.

### Analytical procedure

The main analysis procedures of this study were as follows:

Firstly, descriptive statistics were used to analyze the information of 102 mothers of children with ASD.

Secondly, according to Hayes’s suggestion [45], the SPSS plug-in PROCESS was used to test the mediating role of the five sub-dimensions of children’s behavioral problems (conduct behavior, learning problems, psychosomatic disorders, impulsivity-hyperactivity, and anxiety) in the relationship between parent-child conflict and maternal anxiety. The BOOTSTRAP was used for parameter estimation. The sample size was 5000. The 95% confidence interval didn’t include 0, which meant that the parameter was significant.

Finally, according to Edwards and Lambert [46], parenting stress was divided into high and low stress levels. The moderating effects of high and low stress levels on parent-child conflict, child problem behavior, and maternal anxiety were estimated. In the grouping of maternal parenting stress levels, the high-level group was one standard deviation (SD) above the mean (M + 1SD), and the low-level group was one standard deviation (SD) below the mean (M-1SD).

## Results

### Common method bias test

Since the data in this study, all come from the mother’s evaluation, during the data collection process, anonymous participation, reverse question scoring, and balanced scale order were used for control [47]. Harman’s single factor test was used to conduct unrotated factor analysis for possible common method deviations, and the total number of factors with characteristic roots greater than 1 was found to be 29. The explanation rate of the first common factor was 22.23%, which was less than the critical value of 40%. Therefore, it was believed that the data in this study didn’t have serious common method bias problems.

### Demographic information

Table 1 presents demographic information on mothers of autistic children. The average age of the mothers was 36.13 ± 0.67 years old; a very small number of mothers were divorced or unmarried; more than half of the mothers had a bachelor’s degree or college education; nearly a quarter of the mothers were unemployed; 60.8% of the families had only one child; the gender of the children was 79 boys and 23 girls and the age of the children was mainly 3–6 years old.

**Table 1:** Demographic information (*N* = 102)

Variables		Mean (SD)/(*n*%)
Age		36.130(0.667)
Marital status	Unmarried	3(2.9)
	Married	95(93.1)
	Divorce	4(3.9)
Education	Primary school	7(6.9)
	Junior high school	19(18.6)
	High school	20(19.6)
	Undergraduate/junior college	48(47.1)
	Master’s degree or above	8(7.8)
Occupations	Enterprises	22(21.6)
	Self-employed	8(7.8)
	Teachers, doctors, and other professional and technical personnel	14(13.7)
	Farm	8(7.8)
	Worker	11(10.8)
	Freelancing	16(15.7)
	Unemployed	23(22.5)
Monthly household income	¥1000–1999	7(6.9)
	¥2000–4999	35(34.3)
	¥5000–9999	29(28.4)
	¥10,000–19,999	19(18.6)
	¥20,000 or more	9(8.8)
	I don’t know	3(2.9)
Number of children	1	62(60.8)
	2 or more	40(39.2)
The cost of treatment per month		6931.68(10407.785)
Gender	Boy	79(77.5)
	Girl	23(22.5)
The age of the child	3–6	72(70.6)
	7–8	15(14.7)
	9–10	7(6.9)
	11–12	8(7.8)

### Descriptive statistics and correlations

Table 2 shows the descriptive, correlation, and reliability results regarding the variables of interest. The results showed that the mother’s age was somewhat related to the main study variables. Therefore, it was included as a control variable in the subsequent analyses. Mother’s state anxiety was significantly positively correlated with the dimensions of parent-child conflict, parenting stress, conduct problems in children’s behavioral problems, psychosomatic disorders, and anxiety. Mother’s trait anxiety was significantly positively correlated with the dimensions of parent-child conflict, conduct problems in children’s behavioral problems, psychosomatic disorders, and anxiety. Therefore, in the next test of the mediating effect, the dimensions of conduct problems, psychosomatic disorders, and anxiety in children’s behavioral problems were tested as mediating variables.

**Table 2:** Means, standard deviations and correlations among variables

	1	2	3	4	5	6	7	8	9	10
1.Mother’s age	1									
2.Parent-child conflict	− 0.241^*^	1								
3.Parenting Stress	− 0.108	0.372^***^	1							
4.Conduct problems	− 0.127	0.445^***^	0.443^***^	1						
5.Learning problems	− 0.051	0.218^*^	0.417^***^	0.532^***^	1					
6.Psychosomatic disorders	− 0.256^**^	0.472^***^	0.316^**^	0.771^***^	0.280^**^	1				
7.Impulsive-hyperactive	− 0.070	0.350^***^	0.440^***^	0.622^***^	0.568^***^	0.392^***^	1			
8.Anxiety	− 0.160	0.352^***^	0.401^***^	0.826^***^	0.454^***^	0.698^***^	0.564^***^	1		
9.State anxiety	− 0.086	0.221^*^	0.271^**^	0.221^*^	0.100	0.357^***^	0.077	0.206^*^	1	
10.Trait anxiety	− 0.041	0.280^**^	0.185	0.277^**^	0.031	0.326^***^	0.010	0.208^*^	0.684^***^	1
M	36.130	2.831	2.646	2.044	2.569	1.659	2.382	1.973	2.275	2.147
SD	6.667	0.752	0.834	0.649	0.689	0.721	0.744	0.725	0.479	0.435

*Note* *P < 0.05, **P < 0.01, ***P < 0.001 (the same below)

### Mediation effect test

**Fig. 1:**
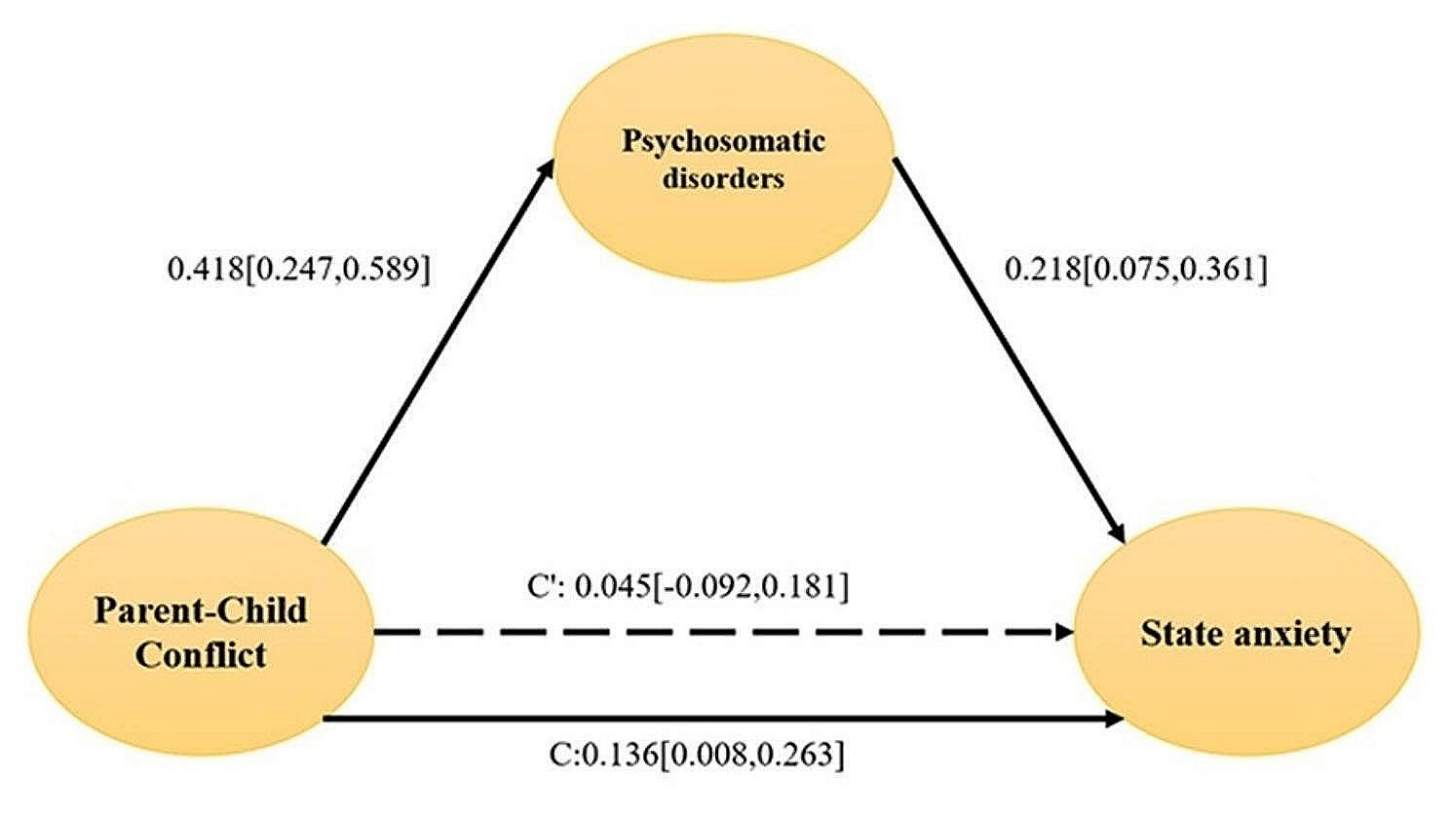
Diagram of the mediating effect of psychosomatic disorders on the impact of parent-child conflict on state anxiety

**Fig. 2:**
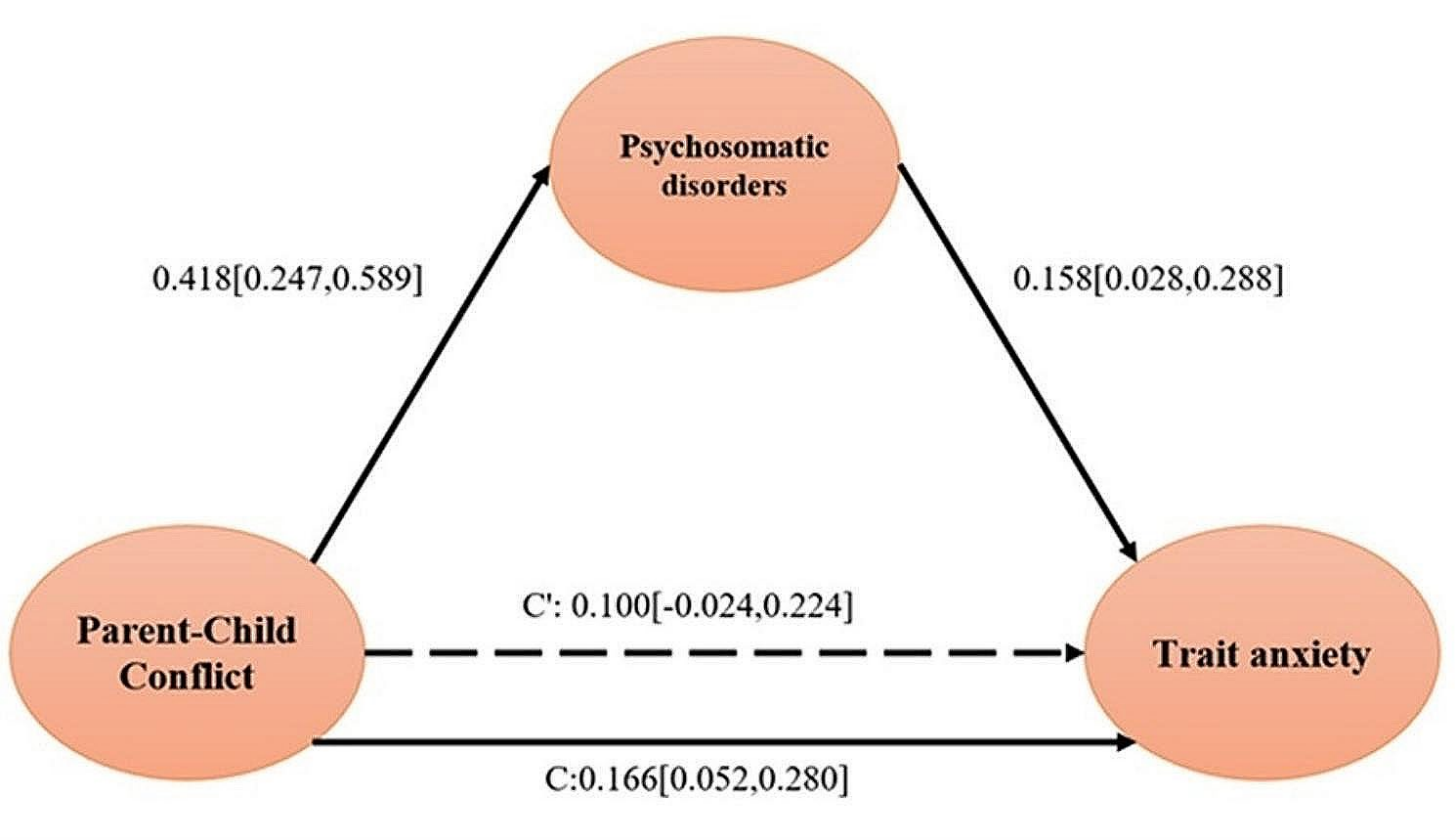
Diagram of the mediating effect of psychosomatic disorders on the impact of parent-child conflict on trait anxiety

Table 3 shows that parent-child conflict positively affected mothers’ state-trait anxiety (B = 0.136, *P* < 0.05; B = 0.166, *P* < 0.01). The results also showed that parent-child conflict was significantly related to psychosomatic disorders in children with ASD (B = 0.418, *P* < 0.001), and psychosomatic disorders in children with ASD were significantly related to mother’s state-trait anxiety (B = 0.217, *P* < 0.01; B = 0.158, *P* < 0.05). Under this condition, the indirect effect of parent-child conflict on state-trait anxiety in mothers of ASD children was significant (B = 0.091, 95% confidence interval [CI]: [0.028, 0.175]; B = 0.066, 95% confidence interval [CI]: [0.004, 0.157]), the mediating effect plot was shown in Fig. 1. and Fig. 2. Children’s conduct problems and anxiety didn’t mediate the impact of parent-child conflict on maternal anxiety. In summary, the severity of children’s psychosomatic disorders fully mediated the relationship between parent-child conflict and state-trait anxiety in mothers.

**Table 3:** Results of mediating hypotheses

	Conduct problems	State anxiety		Trait anxiety	
	Model1	Model2	Model3	Model2	Model3
Constant	1.040(0.443) ^*^	1.864(0.364) ^***^	1.981(0.356) ^***^	1.478(0.323) ^***^	1.611(0.319) ^***^
Age	-0.021(0.009)	-0.032(0.007)	-0.035(0.007)	0.002(0.006)	0.002(0.007)
Parent-Child Conflict	0.441(0.080) ^***^	0.093(0.071)	0.136(0.064)^*^	0.128(0.071)	0.166(0.058)^**^
Conduct problems		0.112(0.080)		0.128(0.071)	
Total effect[95%CL]		0.136[0.008,0.263]		0.166[0.052,0.280]	
Direct effect[95%CL]		0.093[-0.048,0.234]		0.118[-0.008,0.243]	
Indirect effect[95%CL]		0.067[-0.018,0.199]		0.049[-0.002,0.130]	
	Psychosomatic disorders	State anxiety		Trait anxiety	
	Model1	Model2	Model3	Model2	Model3
Constant	1.065(0.478) ^*^	1.749(0.351) ^***^	1.981(0.356) ^***^	1.443(0.319) ^***^	1.611(0.319) ^***^
Age	-0.016(0.010)	0.001(0.007)	-0.003(0.007)	0.004(0.006)	0.002(0.007)
Parent-Child Conflict	0.418(0.086) ^***^	0.045(0.069)	0.136(0.064)^*^	0.100(0.062)	0.166(0.058)^**^
Psychosomatic disorders		0.217(0.072) ^**^		0.158(0.065) ^*^	
Total effect[95%CL]		0.136[0.008,0.263]		0.166[0.052,0.280]	
Direct effect[95%CL]		0.045[-0.09 2 ,0.181]		0.100[-0.024,0.224]	
Indirect effect[95%CL]		0.091 [0.028,0.175]		0.066[0.004,0.157]	
	Anxiety	State anxiety		Trait anxiety	
	Model1	Model2	Model3	Model2	Model3
Constant	1.376(0.516) ^**^	1.850(0.367) ^***^	1.981(0.356) ^***^	1.506(0.329) ^***^	1.611(0.319) ^***^
Age	-0.009(0.011)	-0.002(0.007)	-0.002(0.007)	0.003(0.007)	0.002(0.007)
Parent-Child Conflict	0.321(0.093) ^***^	0.105(0.068)	0.136(0.064)^*^	0.077(0.062)	0.166(0.058)^**^
Anxiety		0.096(0.069)		0.141(0.061) ^*^	
Total effect[95%CL]		0.136[0.008,0.263]		0.166[0.052,0.280]	
Direct effect[95%CL]		0.105[-0.011,0.098]		0.141[0.021,0.262]	
Indirect effect[95%CL]		0.048[-0.017,0.149]		0.025[-0.016,0.085]	

### Moderating effect test

The moderating effect of a mother’s parenting stress is shown in Table 4. The interaction term between parent-child conflict and maternal parenting stress significantly predicted maternal state anxiety (B=-0.005, t=-0.814, *p* < 0.001) and trait anxiety (B=-0.001, t=-0.095, *p* < 0.001). In summary, parenting stress significantly mediated the impact of parent-child conflict on maternal state anxiety and trait anxiety.

To reveal the essence of the interaction, a simple slope analysis was carried out, and the moderating variable parenting stress was added or subtracted by one standard deviation as the high-stress group and the low-stress group respectively.

**Fig. 3:**
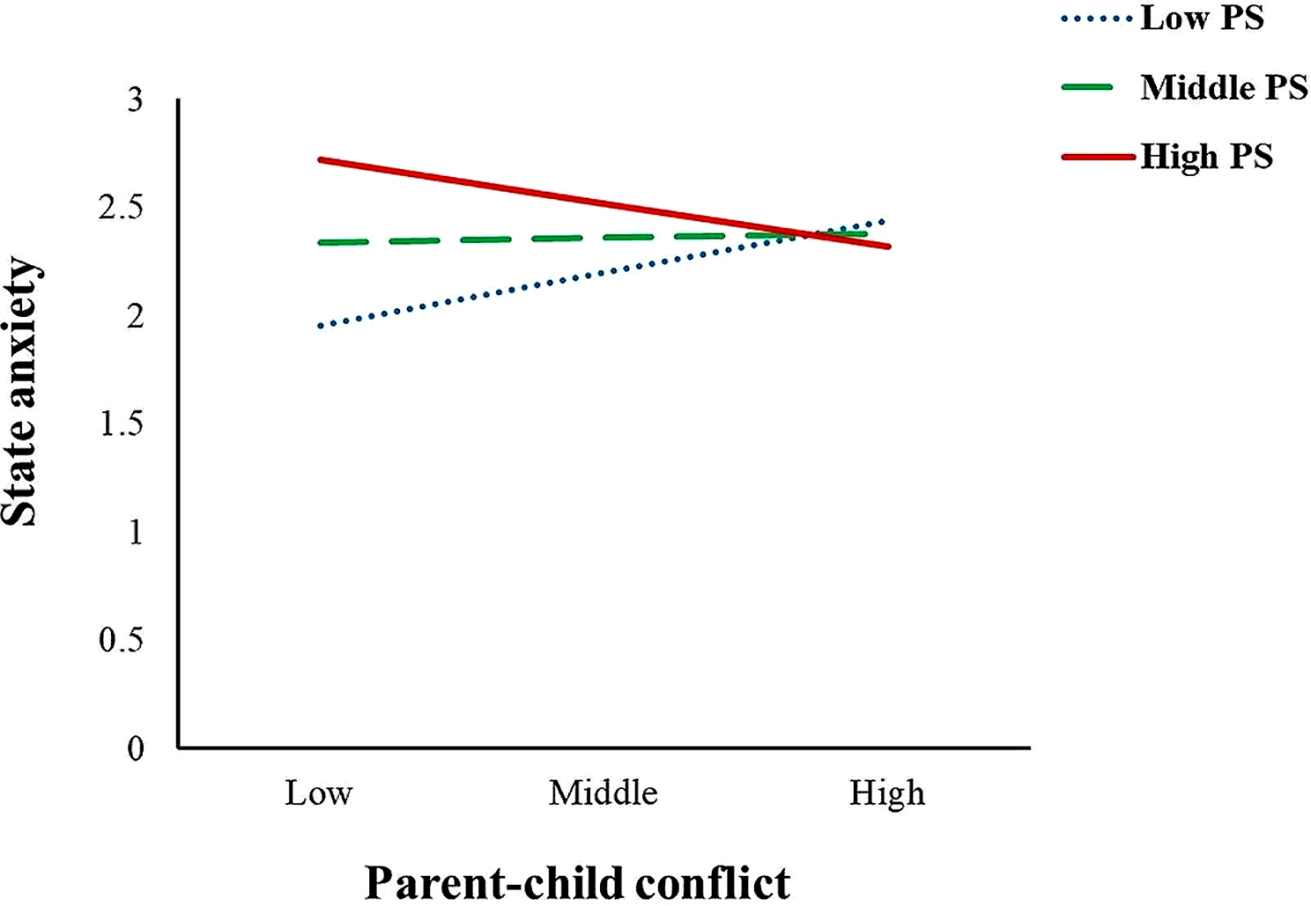
Parenting stress as a moderator between parent-child conflict and STATE anxiety (PS = Parenting Stress, the same as Fig. 4.)

Firstly, parenting stress had a moderating effect on the relationship between parent-child conflict and maternal state anxiety (Fig. 3). The results found that when the level of parenting stress was high, parent-child conflict had a significant negative predictive effect on state anxiety (simple slope=-0.27, t=-2.91, *p* < 0.01); when the level of parenting stress was low, parent-child conflict had a significant positive predictive effect on state anxiety (simple slope = 0.32, t = 4.22, *p* < 0.001), and the predictive effect is large (the value of simple slope changes from − 0.27 to 0.32). The specific performance was as follows: for mothers with high parenting pressure, as parent-child conflict increases, the mother’s state anxiety will decrease; for mothers with low parenting stress, as parent-child conflict increases, their state anxiety will increase significantly.

**Fig. 4:**
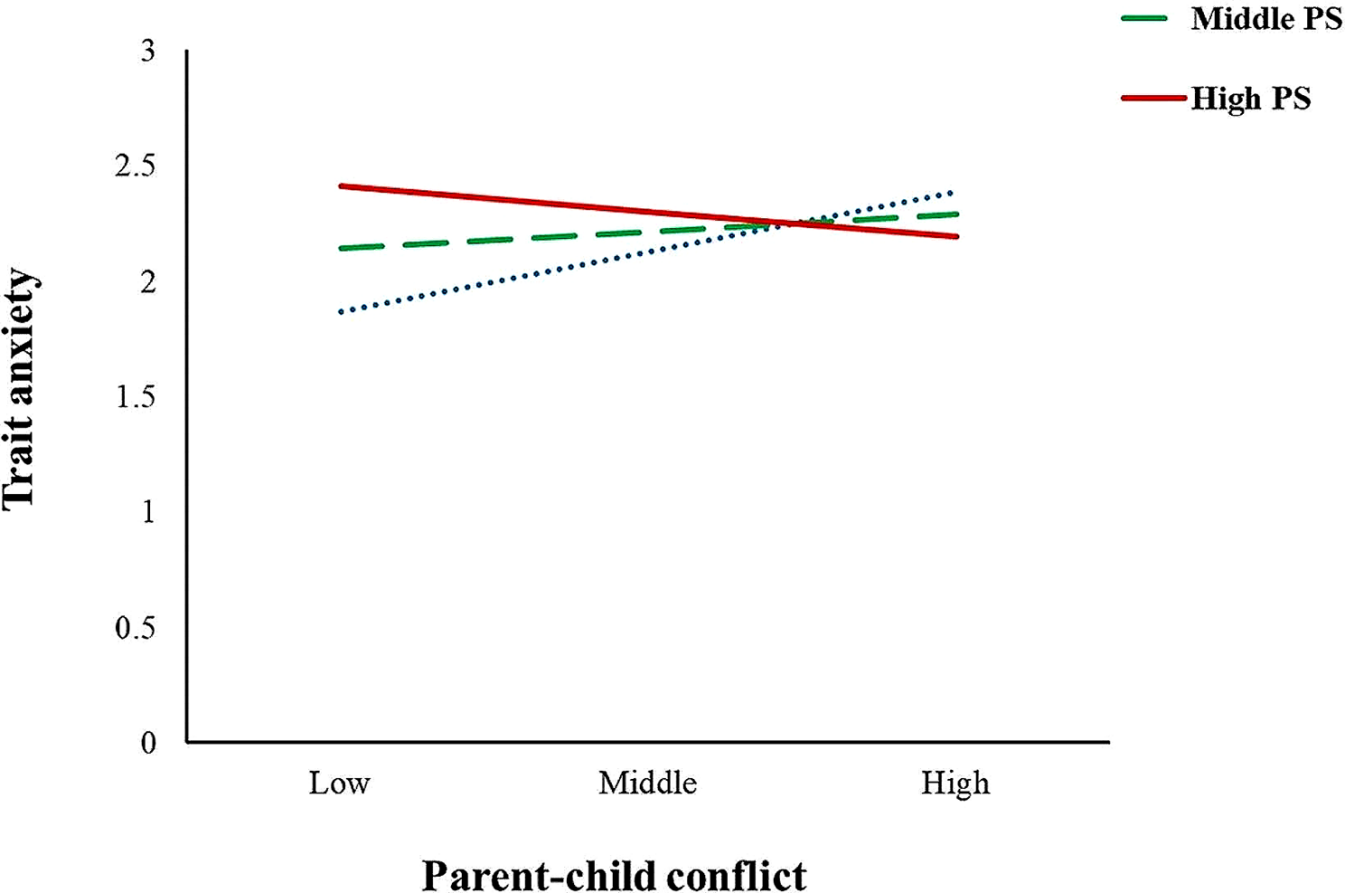
Parenting stress as a moderator between parent-child conflict and TRAIT anxiety

Secondly, parenting stress had a moderating effect on the relationship between parent-child conflict and maternal trait anxiety ((Fig. 4). The results found that when the level of parenting stress was high, the predictive effect of parent-child conflict on maternal trait anxiety was not significant (*p* = 0.093); when the level of parenting stress was low, parent-child conflict had a significant positive predictive effect on state anxiety (simple slope = 0.34, t = 4.80, *p* < 0.001). The performance was as follows: for mothers with high parenting stress, their trait anxiety levels were higher regardless of parent-child conflict; for mothers with low parenting stress, a significant increase in trait anxiety occurred as parent-child conflict increased.

**Table 4:** Results of moderating hypotheses

Result variable	State anxiety			Trait anxiety		
Predictor variable	B	SE	t	B	SE	t
Age	-0.005	0.006	-0.814	-0.001	0.006	-0.095
Parent-Child Conflict	0.027	0.062	0.442	0.098	0.057	1.718
Parenting Stress	0.193	0.055	3.507 ^**^	0.105	0.051	2.052 ^*^
Parent-Child Conflict × Parenting Stress	-0.355	0.070	-5.083 ^***^	-0.293	0.065	-4.502 ^***^
R	0.531			0.495		
R ^2^	0.282			0.245		
F	9.535			7.863		

## Discussion

From the perspective of the family, this study revealed the mechanism of children’s behavioral problems and parenting stress on the relationship between parent-child conflict and maternal anxiety. It showed that alleviating parenting stress and reducing the probability of parent-child conflict had a positive significance for improving the anxiety state of mothers of children with ASD and promoting their mental health development.

### The relationship between parent-child conflict and anxiety in mothers of children with ASD

This study found that parent-child conflict can significantly and positively predict anxiety in mothers of children with ASD, which was similar to previous research results. In a study of children with autism, more than half of the mothers experienced significant psychological distress, and parent-child conflict was one of the predictors [48]. In families of children with ASD, both mother-child and father-child relationships were affected by parents’ mental health [33]. Stress, anxiety, and stress and depressive symptoms in mothers of children with autism predict poorer parent-child relationships [49]. Another study pointed out that compared with families of non-autistic children, parents of autistic children were more likely to experience stress and depression, and these emotions were also related to parent-child conflict [50]. In caring for a child with autism, a mother may find that her own needs and the needs of other family members were ignored or placed on the back burner. Therefore, early interventions that support mothers may help reduce their stress and anxiety to improve families’ overall quality of life [51]. Previous studies generally focused on the relationship between the parent-child relationship and mental health problems of mothers of ASD children. This study further refined the dimension of mental health problems and paid attention to the impact of parent-child conflict on the anxiety of mothers of ASD children.

### The mediating role of children’s behavioral problems

This study found that psychosomatic disorders in children’s behavioral problems play a mediating role between parent-child conflict and anxiety in mothers of children with ASD.

On the one hand, conflictual parent-child relationships can easily lead to serious psychosomatic disorders in children. Parent-child conflicts can affect the quality of parent-child relationships, undermine the stability of family relationships, make children feel insecure, and trigger children’s behavioral questions [17]. Some studies have also shown that the parenting stress caused by children with ASD usually affects the entire family, and the symptoms of children with ASD can have an impact on family relationships (parent-child relationship) [52].

On the other hand, the severity of children’s behavioral problems will have an impact on the anxiety of mothers of children with ASD, which is consistent with existing research results. Behavioral problems in children with autism have a positive predictive effect on parental anxiety [36], children’s behavioral problems trigger sources of stress and anxiety in mothers [53], and the state and trait anxiety of mothers are positively related to the total score of children’s difficulties and emotional symptoms [54]. Therefore, children’s behavioral problems become an important factor in alleviating mothers’ anxiety levels, which also provides evidence for the importance of early intervention for children with autism.

### The moderating role of parenting stress

This study found that parenting stress mediates the relationship between parent-child conflict and maternal anxiety in children with ASD. At low parenting stress levels, parent-child conflict has a greater predictive effect on maternal state and trait anxiety. At high parenting stress levels, the parent-child conflict has a greater predictive effect on maternal state and trait anxiety. The predictive effect of parent-child conflict on maternal trait anxiety was not significant, this is similar to previous research results. Compared with mothers of ordinary children, ASD caregivers face greater parenting stress [55]. High levels of parenting stress and depressive symptoms are associated with maladaptive parenting behaviors and/or related to low-quality parent-child relationships. High parenting pressure can also cause negative parenting behaviors in mothers [56], forming a bad parent-child relationship [57], leading to a decrease in the frequency of parent-child conflicts, resulting in more severe negative emotions [58, 59].

Under low parenting stress levels, parent-child conflict will lead to increased state-trait anxiety in mothers of children with ASD. State anxiety mainly reflects a short-term anxious emotional state, which is directly triggered by environmental stimuli and is closely related to life events and stress [54]; Trait anxiety can be understood as a relatively stable emotional pattern reflected by individuals in the face of dangerous situations in the outside world, with significant individual differences [60]. Therefore, under the condition of low parenting stress levels, mothers of children with ASD will pay more attention to external events (conflict-type interactions between parents and children), thereby increasing their state anxiety level. The daily stress of children with ASD caused by impaired social functions caregiving, rehabilitation support, and financial burdens can put mothers in a chronic state of stress for a long time, leading to an increased risk of trait anxiety [61]. Under high levels of parenting stress, maternal trait anxiety will be at a higher level regardless of parenting stress. Parenting stress will make mothers of children with ASD feel long-term anxiety. At this time, external events (conflict-type interactions between parents and children) have no significant predictive effect on the trait anxiety of mothers of children with ASD. Therefore, we should try to reduce the parenting stress of mothers of children with ASD, so that mothers can better face conflicts with their children, thereby improving their anxiety state and improving their mental health.

### Limitations and future directions

Even though this is the first study to reveal the mechanism of parent-child conflict on maternal anxiety in Chinese children with autism from a family perspective, it also has certain limitations. Firstly, as a cross-sectional study, it cannot explore the causal relationship between variables. In the future, it is necessary to further explore the causal relationship between variables through longitudinal research or experimental research. Secondly, the sample size of girls and the overall sample size are small; in addition, this study focuses only included mothers, and data collection on fathers and comparison of anxiety between parents were not considered. Finally, the data used in this study are mothers’ self-assessments, it only examines the relationship between parent-child conflict, child behavioral problems, parenting stress, and maternal anxiety from the mother’s perspective. Future research can use a variety of assessment methods, such as professional evaluation from a different perspective to obtain more comprehensive and objective research conclusions.

## Conclusions

This study elucidated the mechanism of parent-child conflict on maternal anxiety of autistic children and the intricate interactions therein from the perspective of families. The findings provide strong evidence to support the relationship between these key variables. Specifically, parent-child conflict became an important predictor of maternal anxiety in children with autism. In addition, the mediating role of children’s behavior problems in parent-child conflict and maternal anxiety in autistic children emphasizes the importance of early intervention in autistic children. In addition, this study highlights the relationship between parenting stress and parent-child conflict becoming maternal anxiety in children with autism. In a word, this study has important implications for the clinical practice of early family intervention for children with autism.

## Data Availability

The datasets generated during and analyzed during the current study are available from the corresponding author upon reasonable request.
